# Diagnosis of asthma in symptomatic children based on measures of lung function: an analysis of data from a population-based birth cohort study

**DOI:** 10.1016/S2352-4642(17)30008-1

**Published:** 2017-10

**Authors:** Clare Murray, Philip Foden, Lesley Lowe, Hannah Durrington, Adnan Custovic, Angela Simpson

**Affiliations:** a Division of Infection, Immunity and Respiratory Medicine, Faculty of Biology, Medicine and Health, Manchester Academic Health Sciences Centre, University Hospital of South Manchester NHS Foundation Trust, University of Manchester, Manchester, UK; b Royal Manchester Children's Hospital, Central Manchester University Hospitals NHS Foundation Trust, Manchester, UK; c Imperial College London, London, UK

## Abstract

**Background:**

Concerns have been expressed about asthma overdiagnosis. The UK National Institute of Health and Care Excellence (NICE) proposed a new diagnostic algorithm applying four lung function measures sequentially (ratio of forced expiratory volume in 1 s [FEV_1_] to forced vital capacity [FVC] <70%, bronchodilator reversibility ≥12%, fractional exhaled nitric oxide [FeNO] ≥35 parts per billion, and peak expiratory flow variability >20%). We aimed to assess the diagnostic value of three of the tests individually, and then test the proposed algorithm in symptomatic children.

**Methods:**

We used follow-up data at age 13–16 years from the Manchester Asthma and Allergy Study, a prospective, population-based, birth cohort study. We initially present results for the whole population, then by subgroup of disease. To simulate the situation in primary care, we included participants reporting symptoms of wheeze, cough, or breathlessness in the previous 12 months and who were not on regular inhaled corticosteroids. We used an epidemiological definition of current asthma, defined as all three of physician-diagnosed asthma, current wheeze, and current use of asthma treatment, reported by parents in a validated questionnaire. We assigned children with negative answers to all three questions as non-asthmatic controls. We also measured spirometry, bronchodilator reversibility, and FeNO at follow-up; data for peak expiratory flow variability were not available. We calculated the proportion of participants with a current positive lung function test at each step of the algorithm, and recorded the number of participants that met our definition of asthma.

**Findings:**

Of 1184 children born into the cohort, 772 attended follow-up at age 13–16 years between July 22, 2011, and Nov 11, 2014. Among 630 children who completed spirometry, FEV_1_:FVC was less than 70% in ten (2%) children, of whom only two (20%) had current asthma. Bronchodilator reversibility was positive in 54 (9%) of 624 children, of whom only 12 (22%) had current asthma. FeNO was 35 or more parts per billion in 115 (24%) of 485 children, of whom 29 (25%) had current asthma. Only four of 56 children with current asthma had positive results for all three tests (spirometry, bronchodilator reversibility, and FeNO). Conversely, 24 (43%) of the 56 children with current asthma were negative on all three tests. FEV_1_:fvc (p=0·0075) and FeNO (p<0·0001), but not bronchodilator reversibility (p=0·97), were independently associated with asthma in multivariable logistic regression models. Among children who reported recent symptoms, the diagnostic accuracy of the algorithm was poor.

**Interpretation:**

Our findings challenge the proposed cutoff values for spirometry, the order in which the lung function tests are done, and the position of bronchodilator reversibility within the algorithm sequence. Until better evidence is available, the proposed NICE algorithm on asthma diagnosis should not be implemented in children.

**Funding:**

UK Medical Research Council.

## Introduction

**Figure 1:**
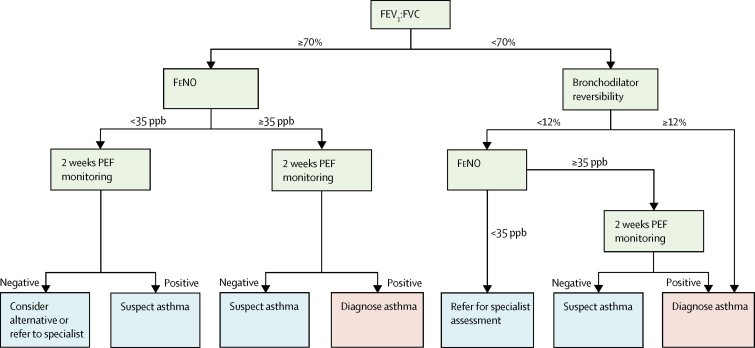
Current NICE criteria for an asthma diagnosis Criteria to be applied in children with symptoms in keeping with asthma. Peak flow should be monitored for 2–4 weeks. More than 20% variability needed for positive test. For patients in whom asthma is suspected, after 6 weeks repeat abnormal tests and review symptoms. NICE=National Institute for Health and Care Excellence. FEV_1_:FVC=ratio of forced expiratory volume in 1 s and forced vital capacity. FeNO=fractional exhaled nitric oxide. PEF=peak expiratory flow. ppb=parts per billion. Figure adapted from NICE guidance for children.^4^

Asthma remains the most common chronic disease of childhood, with 1·1 million children in the UK currently receiving treatment.^1^ Because of the absence of gold standard tests to confirm or refute asthma, most guidelines concur that asthma is a clinical diagnosis based on a characteristic pattern of symptoms and signs in the absence of an alternative explanation.^2^ A careful reassessment of a large Canadian cohort of 613 adults with a recent diagnosis of asthma ruled out the diagnosis in a third of those assessed.^3^ This finding probably represents a combination of asthma remission and overdiagnosis, but in 2% of the population analysed an alternative serious cardiorespiratory disorder was diagnosed. Because of concerns about overdiagnosis (and underdiagnosis), on behalf of the UK National Institute of Health and Care Excellence (NICE), experts have developed comprehensive guidance on the diagnosis of asthma incorporating objective tests, which are yet to be implemented (figure 1).^4^ Despite the accompanying review highlighting the paucity of evidence in children for the use of lung function measures in the diagnosis of asthma (table 1), the interim report^4^ proposes a diagnostic algorithm for use in primary care in children with symptoms. The algorithm incorporates the sequential use of four measures of lung function and inflammation, each applied as a dichotomous variable: first, spirometry (forced expiratory volume in 1 s [FEV_1_] and forced vital capacity [FVC]) expressed as a ratio FEV_1_:FVC; second, bronchodilator reversibility; third, fractional exhaled nitric oxide (FeNO); and fourth, peak flow variability. For both adults and children, spirometry is the first-line investigation; baseline FEV_1_ is not included, but rather the FEV_1_:FVC ratio, with the proposed cutoff for a positive test being an FEV_1_:FVC ratio of less than 70% or of less than the lower limit of normal if this is known for children. Bronchodilator reversibility is done only if the FEV_1_:FVC ratio is less than 70%, and deemed positive if FEV_1_ improves by 12% or more from baseline; children positive to both tests can be diagnosed with asthma. If bronchodilator reversibility is negative, both a FeNO of 35 or more parts per billion and more than 20% variability in peak expiratory flow measured over 2–4 weeks is required to diagnose asthma (ie, three positive tests are needed). For children with normal spirometry (FEV_1_:FVC ≥70%), bronchodilator reversibility testing is not done, but both a FeNO of 35 or more parts per billion and a more than 20% peak flow variability is required for an asthma diagnosis. Children with other combinations of results should be referred to a specialist for opinion, be reviewed in 6 weeks with repeat tests, or have other diagnoses considered.

**Table 1:** Summary of evidence of lung function measures used in the diagnosis of asthma

	**n**	**Sensitivity, % (95% CI)**	**Specificity, % (95% CI)**
**Spirometry**			
FEV_1_:FVC (no studies identified)	··	NA	NA
FEV_1_ <80%^5^	133	52% (··)	72% (··)
**Bronchodilator reversibility**			
No studies identified	··	NA	NA
**FeNO**			
>22 ppb^6^	245	57% (··)	87% (··)
**Peak flow variability**			
Diurnal peak flow variability (mean over 2 weeks >12·3%)^7^	61	50% (30–70)	72% (56–84)

Studies were restricted to those including only children aged 5–16 years, and the table is adapted from the UK National Institute of Health and Care Excellence interim report.^4^ We found some additional studies that included mixed populations of adults and children, but because the paediatric data could not be separated out they were not included here.^8^, ^9^, ^10^ FEV_1_=forced expiratory volume in 1 s. FVC=forced vital capacity. NA=not applicable. FeNO=fractional exhaled nitric oxide. ppb=parts per billion.

**Research in context**

**Evidence before this study**

Asthma is the most common chronic disease in childhood; however, no diagnostic criteria exist to confirm or refute the diagnosis. Guidelines from the British Thoracic Society and Scottish Intercollegiate Guidelines Network recognise that the diagnosis of asthma is a clinical one. Because of concerns about the diagnosis of asthma, particularly overdiagnosis, the UK National Institute of Health and Care Excellence (NICE) has developed comprehensive guidance on the diagnosis incorporating objective tests. The NICE interim report proposes a diagnostic algorithm incorporating the sequential use of four measures of lung function and inflammation in children with symptoms, each applied as a dichotomous variable: spirometry (forced expiratory volume in 1 s [FEV_1_] and forced vital capacity [FVC] expressed as a ratio FEV_1_:FVC), bronchodilator reversibility, fractional exhaled nitric oxide, and peak flow variability. For the development of the NICE guideline, systematic scientific literature searches were done to identify all published evidence relevant to the review questions. The authors acknowledged that no evidence was found for asthma diagnosis in children for either FEV_1_:FVC or bronchodilator reversibility, and little evidence exists for fractional exhaled nitric oxide and peak flow variability. Despite these findings, the guideline proposes a complex algorithm in which at least two of these tests must be positive to make the diagnosis.

**Added value of this study**

To test these proposed algorithms prospectively in newly presenting symptomatic children will take several years. Cohort studies already exist with a wealth of lung function and clinical data and offer an opportunity to quickly assess the likely success of such an algorithm, the sequence, and the proposed cutoffs for the individual tests. In this study, we tested the proposed NICE algorithm using data from a population-based cohort, focusing on adolescents aged 13–16 years with recent asthma symptoms, to simulate the situation in clinical practice. We found poor agreement between the algorithm and our questionnaire-based epidemiological definition of asthma (physician diagnosis, present symptoms, and regular use of inhaled corticosteroids).

**Implications of all the available evidence**

There is wide agreement that an asthma diagnostic pathway incorporating objective measures would be extremely helpful and might subsequently reduce the need for trials of treatment, which can be costly and time consuming. However, evidence so far is clearly insufficient to establish such an algorithm. Our findings challenge the cutoff values defined for spirometry, the order in which the tests are done, and the position of bronchodilator reversibility in the proposed NICE algorithm, which seems to add little in children with symptoms suggestive of asthma. This study makes an important contribution to knowledge in this difficult area. It questions the rationale of the proposed algorithm, which is predominantly based on extrapolated adult data, and highlights the need for an appropriately designed study in the appropriate age group to generate evidence of the value of different tests for the diagnosis of asthma in children.

The accuracy of these tests in this specific sequence for the diagnosis of asthma in children is unknown. Ideally, the proposed algorithms should be tested in prospective studies of newly presenting patients in primary care, which would take several years to complete. Within the Manchester Asthma and Allergy Study, a population-based birth cohort, a wealth of data already exist—including respiratory symptoms, prescribed treatments, and three of the four measures of lung function included in the algorithm—making this a practical setting in which to test a diagnostic algorithm. In this study, we aimed to assess the diagnostic value of each test individually and then test the proposed algorithm using data collected in adolescence, focusing on participants with recent asthma symptoms who were not receiving asthma treatment, to simulate the situation in primary care.

## Methods

### Study design and population

In this analysis, we used data from the Manchester Asthma and Allergy Study,^11^ a population-based birth cohort study done in two centres in Manchester, UK, using data collected at age 13–16 years. Participants were born in the University Hospital of South Manchester (an academic hospital) and Stepping Hill Hospital (a district general hospital), and the study was done at University Hospital of South Manchester. Participants were recruited prenatally and followed up prospectively at intervals of 2–5 years. The local research ethics committee approved the study. Parents provided written informed consent and children gave assent.^11^

The initial analysis presented uses the whole population, then by subgroup of disease. Additionally, we assumed that children without any respiratory symptoms would not be assessed for asthma diagnosis. Therefore, to simulate what might happen in primary care, we tested the proposed NICE algorithm using follow-up data at age 13–16 years from participants who reported respiratory symptoms (wheeze, cough, or breathlessness) in the previous 12 months and who were not on regular inhaled corticosteroids (because these drugs are associated with improved lung function and decreased FeNO).^12^ We calculated the proportion of participants with a positive lung function test at each step of the algorithm, and recorded the number of participants that met our definition of current asthma.

### Data collection

At the age 13–16 years follow-up visit, validated questionnaires^13^ were interviewer-administered in clinic or at home to collect information on parentally reported symptoms, physician-diagnosed diseases, and prescribed treatments. We took spirometry, bronchodilator reversibility, and FeNO measurements.

We defined current asthma as a positive answer to all three of the following questions: “Has the doctor ever told you that your child had asthma?”; “Has your child had wheezing or whistling in the chest in the past 12 months?” and “Has your child had asthma treatment in the past 12 months?” Children whose parents responded negatively to all three questions were defined as non-asthmatic controls. Participants with one or two of the above features, or incomplete data, were defined as having possible asthma. Current rhinitis was defined as a positive response to the question: “In the past 12 months, has your child ever had a problem with sneezing, or a runny nose, or a blocked nose when he/she did not have a cold or flu?”

FeNO was measured^14^ with either a chemiluminescence analyser (NIOX, Solna, Sweden) or an electrochemical analyser (NIOX); chemiluminescence was changed over to an electrochemical analyser on May 4, 2012, with no difference found in recorded results between machines (p=0·74; appendix). Spirometry was measured in the clinic with a pneumotachograph-based system (Jaeger, Wurzberg, Germany) or at home with a flow turbine spirometer (Micro Medical, Basingstoke, UK).^15^ Data were expressed as percentage predicted FEV_1_^16^ and FEV_1_:FVC; we calculated FEV_1_:FVC predicted values and the lower limit of normal.^17^, ^18^ We calculated bronchodilator reversibility as a percentage change after administration of 400 μg of salbutamol with the following equation:

$$\frac{(\text{post}-\text{bronchodilator}\;{\text{FEV}}_{1}-\text{baseline}\;{\text{FEV}}_{1})\times 100}{\text{baseline}\;{\text{FEV}}_{1}}$$

Bronchodilator reversibility was deemed positive if FEV_1_ increased by 12% or more. Peak flow variability was not measured.

### Statistical analysis

We calculated descriptive statistics for diagnostic tests and made comparisons with χ^2^ tests, one-way ANOVA, and Fisher's exact test, as appropriate. Among study participants who met the definition of current asthma or who were non-asthmatic (excluding participants with possible asthma), the variables in the algorithm were assessed with sensitivities, specificities, positive predictive values, negative predictive values, and areas under receiver-operating characteristic curves (AUROCs). We used a multivariable logistic regression model, assuming a linear functional form for the predictors, to investigate the importance of the considered variables. We used Youden's *J* statistic to estimate the best cutoff values of each test individually. We did all analyses with SPSS version 22 and used a 5% significance level throughout.

### Role of the funding source

The funders and sponsors of the study had no role in study design, data collection, data analysis, data interpretation, or writing of the report. The corresponding author had full access to all the data in the study and had final responsibility for the decision to submit for publication.

## Results

Of the 1184 children born into the cohort, 772 attended follow-up at age 13–16 years (mean 15·5 years [SD 0·64]) between July 22, 2011, and Nov 11, 2014 (appendix). Characteristics of the study population are presented in table 2. Comparisons between children included and children excluded in the study, and between male and female participants, are presented in the appendix. Among the 772 participants reviewed, 630 (82%) had measurements for spirometry, 624 (81%) for bronchodilator reversibility, and 485 (63%) for FeNO (table 2); 481 (62%) had all three measurements of lung function, 189 (39%) of whom reported one or more symptom in keeping with possible asthma in the past 12 months. Further descriptions of the lung function parameters are presented in the appendix. For the whole population, measured mean FEV_1_:FVC for girls (89·7% [95% CI 89·0–90·4]) and for boys (86·9% [86·1–87·7]) were very similar to the respective predicted values (89·5% *vs* 86·3%; appendix). Only ten (2%) of 630 children had an FEV_1_:FVC of less than 70% (two with asthma). The mean calculated lower limit of normal for FEV_1_:FVC for girls was 78·2% (95% CI 78·2–78·3) and for boys was 74·8% (74·8–74·9; appendix); 28 (4%) of 630 children had an FEV_1_:FVC below the lower limit of normal, of whom 11 had asthma. An increase after bronchodilator use of FEV_1_ of 12% or more from baseline was seen in 54 (9%) of 624 children (table 2). FeNO was 35 or more parts per billion in 115 (24%) of 485 children, of whom 29 had asthma.

**Table 2:** Characteristics of the study population

		**Whole population**	**Non-asthmatic individuals**	**Individuals with possible asthma**	**Individuals with current asthma**	p value*
**Spirometry**						
Number of patients		630	403	153	74	NA
Sex						
	Male	325 (52%)	193 (48%)	91 (59%)	41 (55%)	0·040†
	Female	305 (48%)	210 (52%)	62 (41%)	33 (45%)	NA
Age (years)		15·6 (15·5–15·6)	15·6 (15·6–15·7)	15·4 (15·3–15·6)	15·4 (15·2–15·6)	0·0021‡
FEV_1_ (L)		3·6 (3·6–3·7)	3·7 (3·6–3·7)	3·7 (3·6–3·9)	3·3 (3·1–3·5)	0·00012‡
FEV_1_ (% predicted)		98·7% (97·7–99·7)	99·3% (98·2–100·4)	100·0% (97·9–102·1)	92·9% (89·6–96·2)	<0·0001‡
FEV_1_:FVC		88·3% (87·7–88·8)	89·2% (88·5–89·8)	87·9% (86·9–89·0)	84·0% (82·3–85·9)	<0·0001‡
FEV_1_:FVC <70%		10 (2%)	5 (1%)	3 (2%)	2 (3%)	0·44§
Currently on regular inhaled corticosteroids		34 (5%)	0	5 (3%)	29 (39%)	··
**Bronchodilator reversibility**						
Number of patients		624	399	151	74	NA
Bronchodilator reversibility		4·9% (4·4–5·3)	4·5% (3·9–5·0)	4·8% (4·0–5·7)	7·2% (5·7–8·8)	0·00043‡
Bronchodilator reversibility ≥12%		54 (9%)	26 (7%)	16 (11%)	12 (16%)	0·015†
**FeNO**						
Number of patients		485	314	115	56	NA
Geometric mean FeNO (ppb)		20·0 (18·8–21·6)	17·4 (16·2–18·8)	22·2 (19·1–25·8)	35·7 (27·3–46·6)	<0·0001‡
FeNO ≥35 ppb		115 (24%)	54 (17%)	32 (28%)	29 (52%)	<0·0001†

Data are n (%) or mean (95% CI), unless stated otherwise. NA=not applicable. FEV_1_=forced expiratory volume in 1 s. FVC=forced vital capacity. FeNO=fractional exhaled nitric oxide. ppb=parts per billion.

* p values compare the three disease groups.

† χ^2^ test.

‡ One-way ANOVA.

§ Fisher's exact test.

We assessed the diagnostic value of each test in children with current asthma (n=74) and without asthma (n=403), excluding children with possible asthma (n=153); for completeness, we included % predicted FEV_1_ (table 3). Our results suggest that values less stringent than those proposed in the NICE algorithm were much more informative (FEV_1_:FVC <83·8%, bronchodilator reversibility ≥3·48%, and FeNO ≥24 parts per billion; table 3).

**Table 3:** Diagnostic values for individual tests in 477 children

	**Sensitivity**	**Specificity**	**Positive predictive value**	**Negative predictive value**
**FEV**_1_**:FVC (n=477; AUROC=0·701)**				
<70%	2/74 (3%)	398/403 (99%)	2/7 (29%)	398/470 (85%)
<75%	8/74 (11%)	391/403 (97%)	8/20 (40%)	391/457 (86%)
<80%	20/74 (27%)	368/403 (91%)	20/55 (36%)	368/422 (87%)
LLN	11/74 (15%)	391/403 (97%)	11/23 (48%)	391/454 (86%)
<83·8%*	40/74 (54%)	328/403 (81%)	40/115 (35%)	328/362 (91%)
**Bronchodilator reversibility (n=473; AUROC=0·636)**				
≥12%	12/74 (16%)	373/399 (93%)	12/38 (32%)	373/435 (86%)
≥15%	7/74 (9%)	380/399 (95%)	7/26 (27%)	380/447 (85%)
≥3·48%*	57/74 (77%)	181/399 (45%)	57/275 (21%)	181/198 (91%)
**FeNO (n=370; AUROC=0·711)**				
≥35 ppb	29/56 (52%)	260/314 (83%)	29/83 (35%)	260/287 (91%)
≥40 ppb	26/56 (46%)	272/314 (87%)	26/68 (38%)	272/302 (90%)
≥24 ppb*	35/56 (63%)	228/314 (73%)	35/121 (29%)	228/249 (92%)
**FEV_1_ % predicted**†**(n=477; AUROC=0·623)**				
<80%	16/74 (22%)	384/403 (95%)	16/35 (46%)	384/442 (87%)
<91·045%*	32/74 (43%)	312/403 (77%)	32/123 (26%)	312/354 (88%)

Data include children with asthma (n=74) and non-asthmatic controls (n=403) who had spirometry. Children with possible asthma were excluded. FEV_1_=forced expiratory volume in 1 s. FVC=forced vital capacity. AUROC=area under the receiver-operating characteristic curve. LLN=lower limit of normal. FeNO=fractional exhaled nitric oxide. ppb=parts per billion.

* The values denote the best cutoffs according to Youden's *J* statistic (sensitivity + specificity − 1) for this population.

† FEV_1_ % predicted does not form part of the diagnostic algorithm but has been included for completeness.

Multivariable logistic regression models revealed that FeNO (p<0·0001) and FEV_1_:FVC (p=0·0075), but not bronchodilator reversibility (p=0·97), were independently associated with asthma (appendix). Each unit increase in FeNO was associated with an odds ratio of 1·03 (95% CI 1·02–1·04) for asthma, whereas each 1% decrease in FEV_1_:FVC was associated with an odds ratio of 1·10 (1·04–1·16). This model had an AUROC of 0·79 (95% CI 0·72–0·86) for predicting asthma, which is higher than the AUROC values for the individual tests (appendix). We found no difference in the magnitude of the effect for FeNO and FEV_1_:FVC when bronchodilator reversibility was removed from the model (appendix).

**Figure 2:**
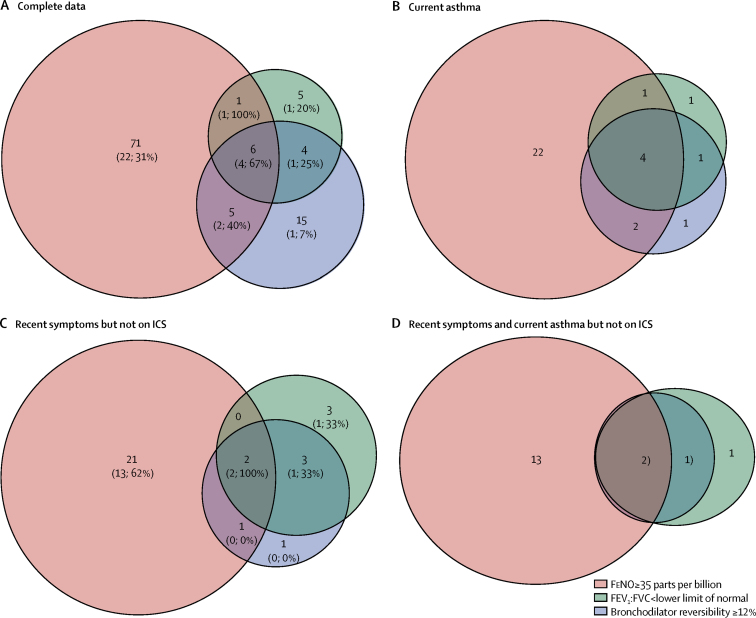
Venn diagrams of overlap between positive tests for spirometry, bronchodilator reversibility, and FeNO in different subgroups of children (A,C) Data are number of patients with positive tests (number of patients with current asthma; percentage with asthma). Patients within the orange circles have a FeNO of 35 or more parts per billion, patients within the green circles have an FEV_1_:FVC of less than the lower limit of normal, and patients within the purple circles have bronchodilator reversibility of 12% or higher. (A) Children with complete data (n=366; 56 with current asthma and 310 without asthma; children with possible asthma excluded; 259 all negative tests [24, 9%] with current asthma). (B) Children with current asthma (n=56; 310 without asthma and children with possible asthma excluded; 24 all negative tests). (C) Children with recent symptoms but not on regular ICS (n=89; 34 with current asthma and 55 without asthma; children with possible asthma excluded; 58 all negative tests [17, 29%] with current asthma). (D) Children with recent symptoms and current asthma who are not on ICS (n=34; 17 all negative tests). FEV_1_:FVC=ratio of forced expiratory volume in 1 s and forced vital capacity. FeNO=fractional exhaled nitric oxide. ICS=inhaled corticosteroids.

Of the 481 children with full lung function data, 56 (12%) had current asthma and 310 (64%) did not have asthma (figure 2); 115 (24%) with possible asthma were excluded. Only six children (four with asthma) had positive results for all three tests (spirometry, bronchodilator reversibility, and FeNO). Conversely, 24 (43%) of the 56 children with asthma were negative on all three tests.

A total of 189 children reported one or more respiratory symptoms within the previous 12 months, of whom 26 received regular inhaled corticosteroids and were excluded from this analysis. Of the remaining 163 children, 34 had current asthma, 55 were classified as non-asthmatic controls (reported cough or breathlessness, but not wheeze), and 74 had possible asthma (excluded from this analysis). The predictive values of each test in this population are shown in table 4 and the appendix.

**Table 4:** Diagnostic values for individual tests in 163 children reporting one or more respiratory symptoms

	**Sensitivity**	**Specificity**	**Positive predictive value**	**Negative predictive value**
**FEV_1_:FVC (n=89; AUROC=0·616)**				
<70%	0/34 (0%)	53/55 (96%)	0/2 (0%)	53/87 (61%)
<75%	2/34 (6%)	51/55 (93%)	2/6 (33%)	51/83 (61%)
<80%	8/34 (24%)	48/55 (87%)	8/15 (53%)	48/74 (65%)
LLN	4/34 (12%)	51/55 (93%)	4/8 (50%)	51/81 (63%)
<85·5%*	19/34 (56%)	38/55 (69%)	19/36 (53%)	38/53 (72%)
**Bronchodilator reversibility (n=89; AUROC=0·594)**				
≥12%	3/34 (9%)	51/55 (93%)	3/7 (43%)	51/82 (62%)
≥15%	2/34 (6%)	52/55 (95%)	2/5 (40%)	52/84 (62%)
≥3·2%*	27/34 (79%)	23/55 (42%)	27/59 (46%)	23/30 (77%)
**FeNO (n=89; AUROC=0·618)**				
≥35 ppb	15/34 (44%)	46/55 (84%)	15/24 (63%)	46/65 (71%)
≥40 ppb	13/34 (38%)	49/55 (89%)	13/19 (68%)	49/70 (70%)
≥37 ppb*	15/34 (44%)	47/55 (85%)	15/23 (65%)	47/66 (71%)
**FEV_1_ % predicted**†**(n=89; AUROC=0·555)**				
<80%	3/34 (9%)	51/55 (93%)	3/7 (43%)	51/82 (62%)
<106·5%*	27/34 (79%)	18/55 (33%)	27/64 (42%)	18/25 (72%)

Data include symptomatic children not using regular inhaled corticosteroids with asthma (n=34) and non-asthmatic controls (n=55). Children with possible asthma were excluded. FEV_1_=forced expiratory volume in 1 s. FVC=forced vital capacity. AUROC=area under the receiver-operating characteristic curve. LLN=lower limit of normal. FeNO=fractional exhaled nitric oxide. ppb=parts per billion.

* These values are the best cutoffs according to Youden's *J* statistic (sensitivity + specificity − 1).

† FEV_1_ % predicted does not form part of the diagnostic algorithm but has been included for completeness.

In the multivariable logistic regression analysis (appendix), the only independent predictor of asthma was FeNO; for each unit increase in FeNO, there was an odds ratio for asthma of 1·02 (95% CI 1·01–1·04; p=0·0062). This model had an AUROC of 0·68 (95% CI 0·57–0·80) for predicting asthma. When bronchodilator reversibility was removed, FeNO remained the only independent predictor of asthma, with a non-significant trend for FEV_1_:FVC (p=0·096; appendix).

Among 89 children with recent symptoms but not on regular inhaled corticosteroids (34 with asthma and 55 without asthma), only two children were positive for all three tests, both of whom had asthma (figure 2). Of 21 participants in this population with only a positive test for FeNO, 13 (62%) had asthma. 17 (50%) of 34 children with asthma and not on regular inhaled corticosteroids were negative on all three tests (figure 2).

**Figure 3:**
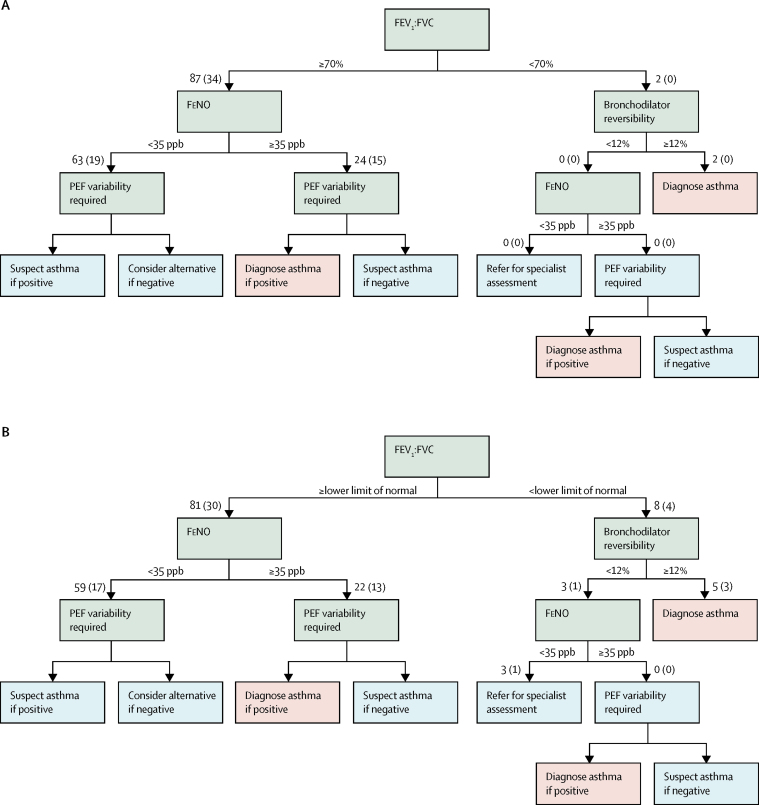
Diagnostic algorithm for 89 children with current symptoms not on inhaled corticosteroids The number in parenthesis denotes the number of children with this test result who had asthma, according to our questionnaire-based definition. PEF data were not available in this population. Obstructive spirometry denoted with an FEV_1_:FVC of less than 70% (A) and of less than the lower limit of normal (B). FEV_1_:FVC=ratio of forced expiratory volume in 1 s and forced vital capacity. FeNO=fractional exhaled nitric oxide. PEF=peak expiratory flow. ppb=parts per billion.

We passed these 89 children with symptoms but not on regular inhaled corticosteroids (34 with current asthma, 55 without asthma) through the sequential NICE diagnostic algorithm, stopping when a diagnosis was reached or the next test (peak flow variability) was unavailable (figure 3). Only two children had an FEV_1_:FVC of less than 70% (figure 3); both had bronchodilator reversibility of 12% or higher, meeting the algorithm criteria for asthma. Neither of these children had current asthma, and FeNO was less than 35 parts per billion in both cases. Of the remaining 87 children with an FEV_1_:FVC of 70% or higher, 24 had a FeNO of 35 or more parts per billion and would be diagnosed with asthma or suspected asthma, depending on the results of peak expiratory flow monitoring. Of these 24 children, 15 had current asthma and nine were non-asthmatics. FeNO was less than 35 parts per billion in the remaining 63 children (19 with current asthma). If peak expiratory flow diaries had shown 20% reversibility for 4 days over 2 weeks in these children, they would fall into the suspect asthma part of the algorithm, in which case tests should be repeated at 6 weeks. For completeness, we repeated this analysis with an FEV_1_:FVC of less than the lower limit of normal (figure 3, appendix) and for all symptomatic children (n=163), including those with possible asthma (appendix).

## Discussion

Using data from a population-based birth cohort, we tested the NICE asthma diagnostic algorithm based on four measures of lung function, which has been proposed for use in primary care. We found poor agreement between the algorithm and our questionnaire-based epidemiological definition of current asthma (physician diagnosis, current symptoms, and current medication) in adolescents aged 13–16 years. In particular, our findings challenge the cutoff values defined for spirometry, the order in which the tests are done, and the position of bronchodilator reversibility within the algorithm sequence, which seems to add little when tested in this population.

Among symptomatic children who had completed three of the four tests, we were able to securely diagnose asthma in only two children using the algorithm, neither of whom met our epidemiological definition; this number increased to five if we used the lower limit of normal rather than less than 70% as the cutoff for FEV_1_:FVC. Almost all other children would require an additional 2 weeks of peak flow monitoring for asthma to be diagnosed or suspected. The economic evaluation of peak flow variability assumes a manual diary will be returned to a practice nurse who will do the calculation (>20% variability on ≥3 days) within 10 min.^4^ Results from a previous study^19^ showed large discrepancies between values recorded electronically and those transcribed into a diary. For peak flow variability to be useful, an electronic peak flow meter (which calculates the variability) will probably be required, changing the economic model.

The proposed algorithm always starts with an FEV_1_:FVC assessment, with a cutoff of 70% (or lower limit of normal in children if available) required to diagnose obstruction. Within our population-based sample, a finding of an FEV_1_:FVC of less than 70% was rare, occurring in only ten (2%) of 630 children, most of whom did not have current asthma. Although we identified additional children with obstructed spirometry (28 [4%] of 630) by replacing the FEV_1_:FVC cutoff of less than 70% with a cutoff of less than the lower limit of normal, we found that the best cutoff for FEV_1_:FVC was much higher at 85·5%, which produced a sensitivity of 56% (19 of 34 participants; compared with a sensitivity of 0% [0 of 34] for FEV_1_:FVC <70%). The lower limit of normal for FEV_1_:FVC falls with age and differs between the sexes, only reaching 70% for most adults by around age 50 years, suggesting that a 70% cutoff is too low for children (appendix).^17^ We recognise that it would not be practical to suggest different cutoff values for boys and girls for each age within a diagnostic algorithm, unless it was fully computerised. In the interim, it would seem sensible to place greater emphasis on the use of the lower limit of normal value, and recommend that this value is made available on spirometers used in primary care. We note that the national guidelines of some countries recommend much higher FEV_1_:FVC values to indicate obstruction (eg, Canada recommends an FEV_1_:FVC of 80%).^20^ Use of FEV_1_:FVC as the initial screen for adults is necessary because the differential diagnosis includes chronic obstructive pulmonary disease and interstitial lung disease, but these diseases are unlikely to be relevant in children.

For those with obstructed spirometry, the next test in the algorithm is bronchodilator reversibility. In our population-based sample, bronchodilator reversibility was seen in 54 (9%) of 624 children, most of whom did not have obstructive spirometry. However, following the diagnostic algorithm in children with symptoms, only eight children would proceed to bronchodilator reversibility (five with positive results, of whom three had current asthma). In children with symptoms of asthma, a cutoff of 12% had only 9% (three of 34 participants) sensitivity for current asthma; the highest sensitivity (79·4%; 27 of 34) was seen for a cutoff of 3·2% (lower than the mean value in this population). Bronchodilator reversibility was not significantly associated with current asthma in the multivariable analysis, perhaps reflecting the negative correlation with FEV_1_:FVC (appendix). Overall, bronchodilator reversibility was less informative than other tests for the diagnosis of current asthma.

FeNO was positive (≥35 parts per billion) in almost a quarter of all children and was the most sensitive test (44% [15 of 34 children]) among children with symptoms. Results from a previous study^6^ in symptomatic children suggested a cutoff of 22 parts per billion, when asthma was diagnosed on the basis of a positive methacholine challenge or bronchodilator reversibility; our data suggested the best cutoff was 37 parts per billion. When we followed the proposed algorithm, FeNO needed to be measured in more than 90% of children. Lung function guidelines of the American Thoracic Society and European Respiratory Society^14^ recommend FeNO be measured before spirometry in adults because spirometry can reduce FeNO by up to 25% in patients both with and without asthma.^21^, ^22^ Considering that FeNO has the best diagnostic accuracy for current asthma, that within the present algorithm almost all children needed FeNO measurement, and that unless done first the value might be an underestimate, we think that measuring FeNO first in children would seem logical.

The ideal setting in which to test the proposed diagnostic algorithm would be a prospective study of patients with newly presenting symptoms who undergo lung function testing while acutely symptomatic before the introduction of any treatment. The outcome of such a study is unlikely to be known within 5 years. Therefore, recognising the paucity of population-based paediatric lung function data in the public domain and the opportunity to test the algorithm in children with both lung function measurements and parent-reported symptom data, we did this assessment using data from our birth cohort. Our study relied on a physician diagnosis of asthma, without details of how this was ascertained. However, we believe that physician diagnosis, together with information on ongoing symptoms and prescribed drugs, is as robust a definition of current asthma that we could achieve in epidemiology. We also took the measurements of lung function at routine visits, without specifically waiting until the child was acutely symptomatic. However, in clinical practice, the general practitioner is unlikely to perform lung function tests when a patient presents with acute symptoms during the consultation. Additionally, because peak flow variability had not been measured prospectively in this birth cohort, we had only data for three of the four possible tests available to analyse. We note that maternal smoking in pregnancy was more common in participants who did not attend follow-up, and it remains possible, although unlikely, that inclusion of such children would have improved the performance of the algorithm. We acknowledge that some of the children who were included as non-asthmatic controls could have had asthma at a younger age. Therefore, we repeated the analysis with these 13 children excluded and found the results were not materially different (data available on request).

Asthma is an umbrella term that includes several phenotypes (which are better characterised in adults than in children); however, at present, the diagnosis is made before any phenotyping and treatment pathways are not dependent on phenotyping for most children with mild-to-moderate asthma. Diagnostic algorithms might need to allow for different patterns of lung function to capture all types of asthma. We recognise that we have tested this algorithm in a population of adolescents (aged 13–16 years) and that this age range is not fully representative of the paediatric population. Results in younger children might reveal different strengths and weaknesses of this algorithm and future prospective studies should aim to include children from age 5–6 years upwards.

We believe that our study makes an important contribution to knowledge in this difficult area. Our findings suggest an urgent need exists for appropriately designed studies to generate evidence about the value of different tests for the diagnosis of paediatric asthma in primary care. Until such evidence is available, the proposed NICE guidance on asthma diagnosis should not be implemented in children.
