# Supplementary material for: Diagnosis of asthma in symptomatic children based on measures of lung function: an analysis of data from a population-based birth cohort study
Source: Lancet Child Adolesc Health. 2017 Oct;1(2):114–23. doi: 10.1016/S2352-4642(17)30008-1 (PMC5616137; doi:10.1016/S2352-4642(17)30008-1)
Supplement: Supplementary appendix [file mmc1.pdf]

THE LANCET
Child & Adolescent Health

Supplementary appendix

This appendix formed part of the original submission and has been peer reviewed. We post it as supplied by the authors.

Supplement to: C Murray, Foden P, Lowe L, et al. Diagnosis of asthma in symptomatic children based on measures of lung function: an analysis of data from a population-based birth cohort study. *Lancet Child Adolesc Health* 2017; published online July 12. http://dx.doi.org/10.1016/S2352-4642(17)30008-1.

Supplementary appendix to [Diagnosing Asthma in Symptomatic Children Using Lung Function: Evidence from a Birth Cohort Study]

Supplementary methods

Spirometry

Dynamic lung volumes and expiratory flow were measured by a heated pneumotachograph system (Jaeger, Germany) or, in the case of home visits, a portable flow turbine based system (Micro Medical, UK) according to the guidelines of the American and European Thoracic Societies. All measurements were made in a sitting position with a nose-clip. The subject was asked to inhale as deeply as possible i.e. to total lung capacity (TLC), then instructed to perform a forced expiration, through a mouthpiece, as hard and as fast as possible until no further gas could be exhaled i.e. to residual volume (RV). The test was repeated at intervals of 30 seconds until 3 technically acceptable traces were obtained and the highest FEV1 was recorded.

FeNO (*Fractional exhaled nitric oxide*)

FeNO was only measured on those attending for follow up within the hospital setting and was not measured on those who had a home visit (n= 107). A further 24 children could not maintain an adequate flow rate to complete the test and 12 missing tests were due to equipment failure.

Exhaled NO measurements were performed using a chemiluminescence analyser (NIOX ;Aerocrine, Sweden) and an electrochemical analyser (NIOX Mino, Aerocrine, Sweden), according to the guidelines of the American and European Thoracic Societies. The subjects were asked to exhale to residual volume and subsequently inhale NO-free air (containing <5ppb) through a filter, to total lung capacity. The subjects were then instructed to perform a single slow expiration through the mouthpiece. Exhalation is carried out against a fixed expiratory resistance, with subjects asked to maintain a positive mouthpiece pressure, to elevate the soft palate closing the velopharyngeal aperture in order to avoid contamination with nasal NO. Subjects were encouraged to maintain a steady and constant flow rate of 0.05 l/s (±10%) with the aid of an exhalation flow display incorporating incentive animation software, which displays a target flow rate or mouthpiece pressure (balloon or meter). An exhalation is deemed adequate if the mean exhalation flow rate is 0.05 l/s (±10%) during the time of the NO plateau generation, and instantaneous flow rate is not <0.045 l/s or >0.055 l/s at any time during the exhalation. Subjects were instructed to exhale for 10 seconds (exhaled volume of at least 0.3l) to allow the airway compartment to be washed out and a reasonable plateau achieved. NO values were measured at the plateau of the end-expiratory reading (over a 3-second window of the expiratory profile) and are expressed as parts per billion (ppb, equivalent to nanoliters per liter). The mean of 3 technically acceptable readings, agreeing within 10% of each other, was recorded (or the reason for measurement failure).

The change in machine occurred in May 2012, and was necessary because the chemiluminescence analyser went out of service.

There was no statistically significant difference in FeNO between those measured using the NIOX (Aerocrine, Sweden) in the early part of the study and the NIOX MINO (Aerocrine, Sweden) used in the later part of the study (NIOX: n=110, GM 20.47ppb, SD (of log transformed values) 0.83, 95% CI 17.49-23.96ppb; NIOX MINO: n=375, GM 19.91ppb, SD (of log transformed values) 0.77, 95% CI 18.41-21.53ppb; p=0.74, independent samples t-test).

Although it appears that the variability is different between the analysers due to the differing width of the 95% CIs, this is the result of the different number of measurements for the analysers. Levene's test for the equality of variances was not statistically significant (p=0.29).

Figure 1. No difference in FeNO levels measured using different methodologies

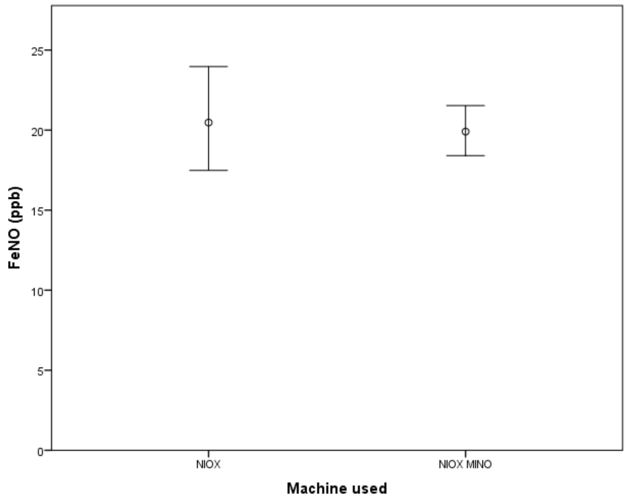

Selection of children with recent symptoms.

Children were defined as having recent symptoms if they answered yes to any of

In the past 12 months has your child had wheezing or whistling in the chest?

Does your child usually have a cough during the day apart from with colds?

Does your child usually have a cough at night apart from with colds?

Does your child cough after exertion apart from with colds?

Does your child cough when he / she is excited apart from with colds?

Does your child cough on exposure to cold air apart from with colds?

Of the 481 children with three measures of lung function, 189 had symptoms of either cough or wheeze in the previous 12 months. The distribution of symptoms is shown in Table 1

Table 1

	No Recent wheeze	recent wheeze	total
No cough	292	39	331
Any cough	83	67	150
total	375	106	481

Of the 26 on regular ICS who were excluded from some analysis, 18 reported both cough and wheeze and 8 reported wheeze in the last 12 months.

Sensitisation

We ascertained sensitisation to allergens by skin prick testing (house dust mite [*Dermatophagoides pteronyssinus*], cat, dog, grasses, moulds, milk, peanut and egg [Bayer, Elkahrt, IN, USA]). Sensitization was defined as a mean wheal diameter of 3 mm or greater than that elicited by the negative control

Statistics

Descriptive statistics were calculated for the main diagnostic tests in the group of patients with spirometry available (N=630). Frequencies and percentages were used to summarise binary or categorical variables and comparisons were made using chi-squared tests. Means, standard deviations and 95% confidence intervals (CIs) were calculated for normally distributed, continuous variables and comparisons were made using independent samples t-tests. FeNO was log normally distributed; results are presented as geometric mean (GM) and 95% CI. Exploratory analyses investigated whether rhinitis affected FeNO (one-way ANOVA, appendix).

The number of children with values above the cut-offs for each of the diagnostic tests considered in the algorithm were presented along with whether they met our definition of asthma. Due to the expected differences between boys and girls, results are presented separately where appropriate. Independent samples t-tests and chi-squared tests were used to compare variables of interest between the genders. Relationships between the variables considered in the algorithm were assessed using correlations.

In the group of children that met our definition of asthma or were non-asthmatic (excluding those with possible asthma), the variables in the algorithm were assessed using sensitivities, specificities, positive predictive values (PPVs), negative predictive values (NPVs), and areas under receiver operating characteristic curves (AUROCs). A multivariable logistic regression model, assuming a linear functional form for the predictors, was used to investigate the importance of the considered variables. This analysis was repeated in the subgroup of symptomatic children not on regular ICS with all required lung function tests available. The diagnostic algorithm was tested in this subgroup of children to approximate its ability when being used in the clinical setting.

All analyses were performed using SPSS 22 and a 5% significance level was used throughout the paper.

Supplementary results

Figure 2. Flow of subjects through study

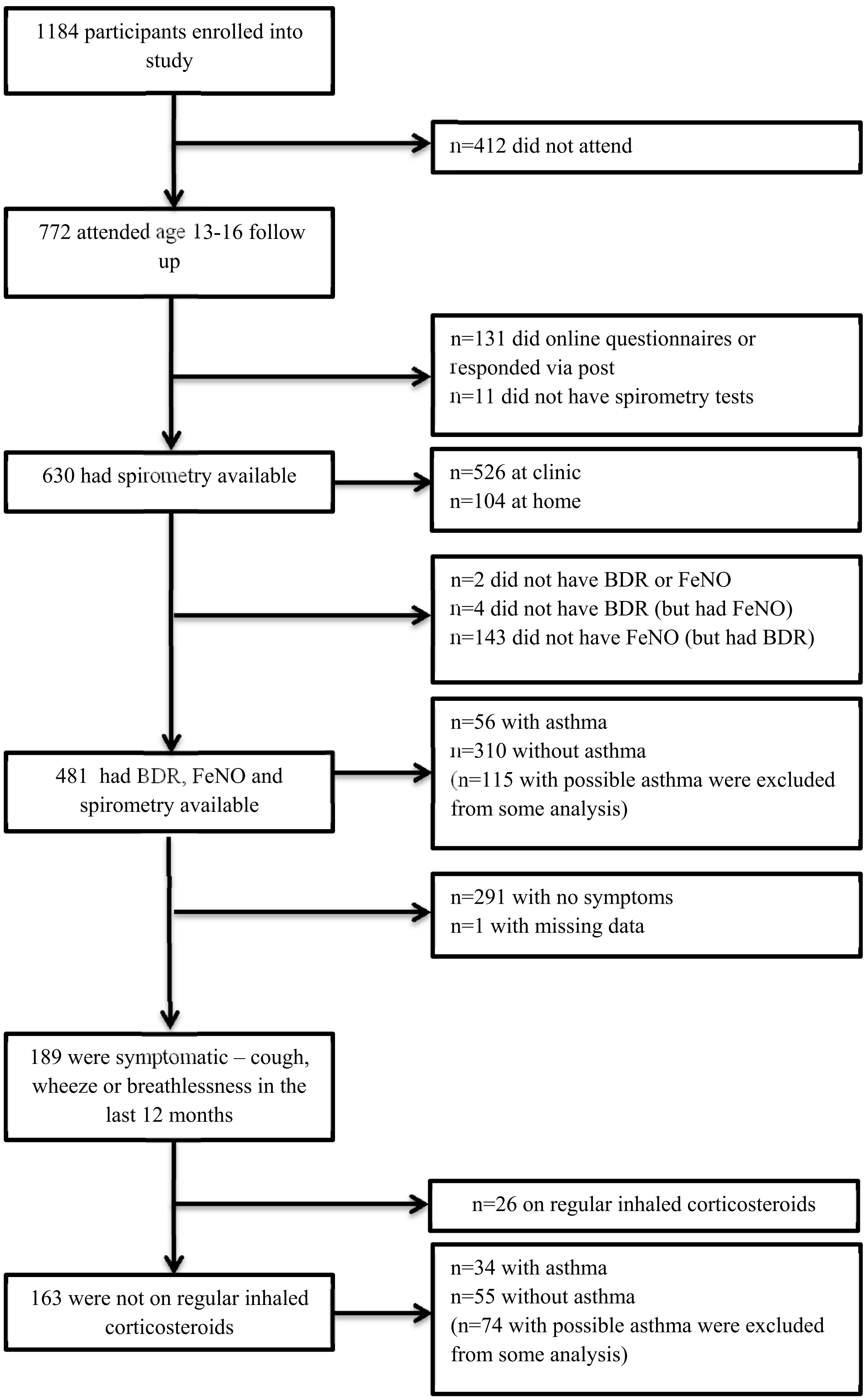

Table 2. Demographic characteristic of children with or without follow up data at age 13-16 years

	Included in study (N=630)	Excluded from study (N=554)	
	n (%)	n (%)	p-value
Gender (Male)	325 (51.6%)	317 (57.2%)	0.052
Maternal smoking (pregnancy)	71/628 (11.3%)	103/549 (18.8%)	<0.001
Maternal asthma (ever)	126 (20.0%)	109 (19.7%)	0.89
Maternal asthma (current)	93 (14.8%)	79/549 (14.4%)	0.86
Paternal asthma (ever)	99 (15.7%)	64/552 (11.6%)	0.040
Paternal asthma (current)	55/627 (8.8%)	30/549 (5.5%)	0.029
Maternal atopy	371/611 (60.7%)	311/535 (58.1%)	0.37
Paternal atopy	396/611 (64.8%)	320/526 (60.8%)	0.17

Table 3. Comparison of lung function and symptoms between males and females

	Boys (N=325)	Girls (N=305)	p-value
FEV_1 (l, mean and 95% CI)	4.05 (3.97-4.12)	3.18 (3.13-3.23)	<0.001[1]
FEV_1(% predicted, mean and 95% CI)	99.52 (98.14-100.91)	97.87 (96.49-99.26)	0.099[2]
FEV_1/FVC (%, mean and 95% CI)	86.91 (86.12-87.69)	89.73 (89.01-90.45)	<0.001[1]
BDR (%, mean and 95% CI)	n=321<br>5.14 (4.56-5.73)	n=303<br>4.58 (3.91-5.24)	0.21[2]
FeNO (ppb, geometric mean and 95% CI)	n=250<br>22.51 (20.45-24.79)	n=235<br>17.70 (16.00-19.57)	0.001[2]
Doctor diagnosis of asthma ever	118/320 (36.9%)	74/301 (24.6%)	0.001[3]
Non-asthmatic<br>Possible asthma<br>Asthma	193 (59.4%)<br>91(28.0%)<br>41 (12.6%)	210 (68.9%)<br>62 (20.3%)<br>33 (10.8%)	0.040[3]
Wheezing in last 12 months	60/319 (18.8%)	50/299 (16.7%)	0.50[3]
Current asthma medication	58 (17.8%)	47 (15.4%)	0.41[3]
Currently on regular ICS	20 (6.2%)	14 (4.6%)	0.39[3]
Any symptoms of cough, wheeze or breathlessness in last 12 months	137 (42.2%)	108/304 (35.5%)	0.089[3]

[1]Independent samples t-test (unequal variances)
[2]Independent samples t-test (equal variances)
[3]Chi-squared test

There was a moderate negative correlation between BDR and FEV_1/FVC (Figure 3a, r=-0.50, p<0.001, rho=-0.46, MIC (maximal information coefficient)=0.27), no correlation between FeNO and FEV_1/FVC (Figure 3b, r=-0.10, p=0.03, rho=-0.11, MIC=0.16), and no correlation between FeNO and BDR (Figure 3c, r=0.06, p=0.22, rho=0.10, MIC=0.19). A MIC is around 0.18 for a random relationship between two variables[1], suggesting there was a moderate relationship between BDR and FEV_1/FVC and no apparent relationship between FeNO and FEV_1/FVC nor FeNO and BDR.

Figure 3. Correlation between measures of lung function and airway inflammation for the whole population. Dotted lines show the algorithm cut-off values.

a) FEV_1/FVC correlated with BDR; upper left quadrant identifies those positive for both tests

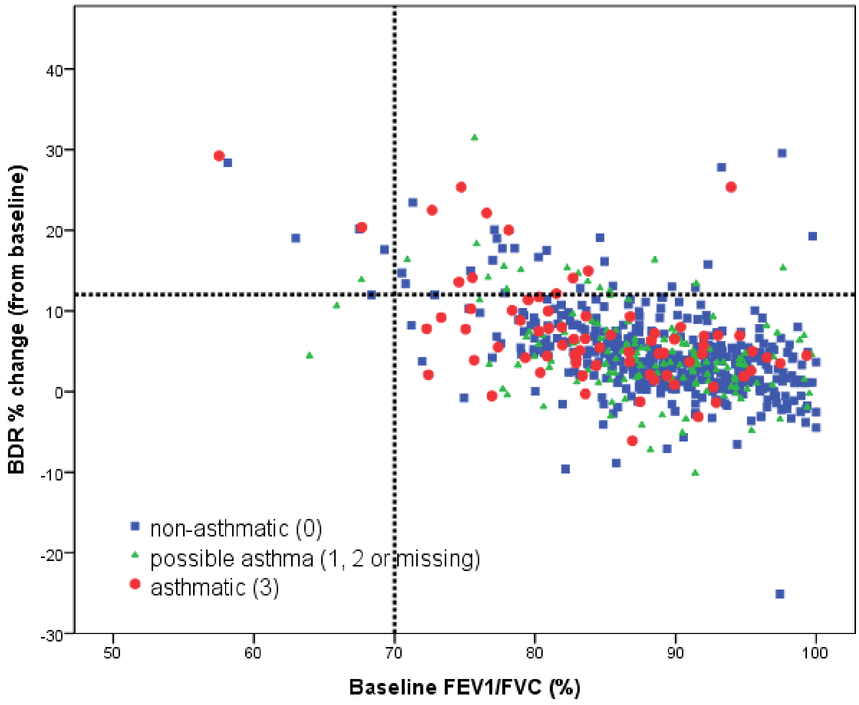

b) FEV_1/FVC correlated with FeNO (Ln); upper left quadrant identifies those positive for both tests

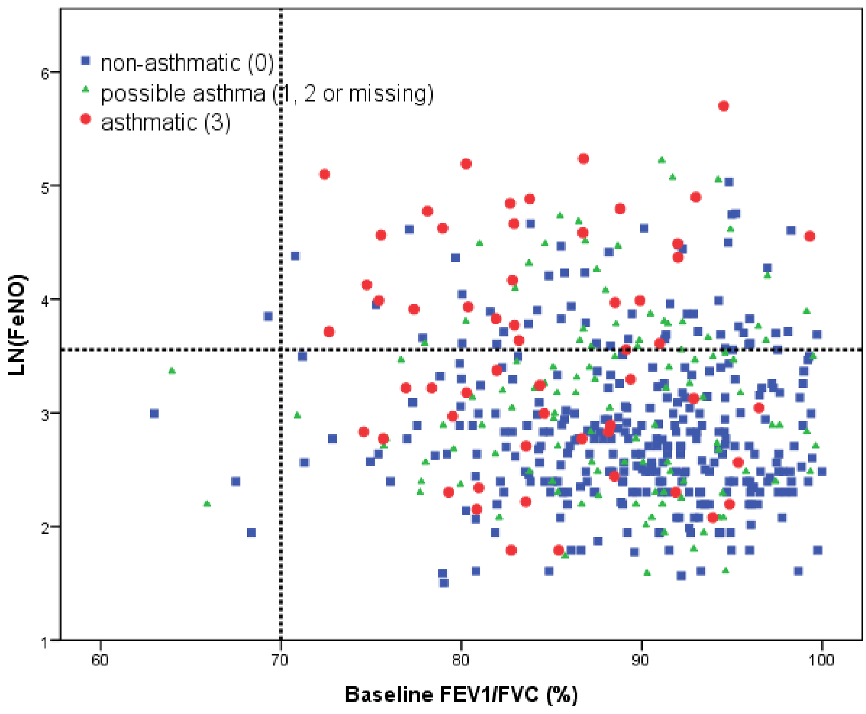

c) BDR correlated with FeNO (Ln); upper right quadrant identifies those positive for both tests

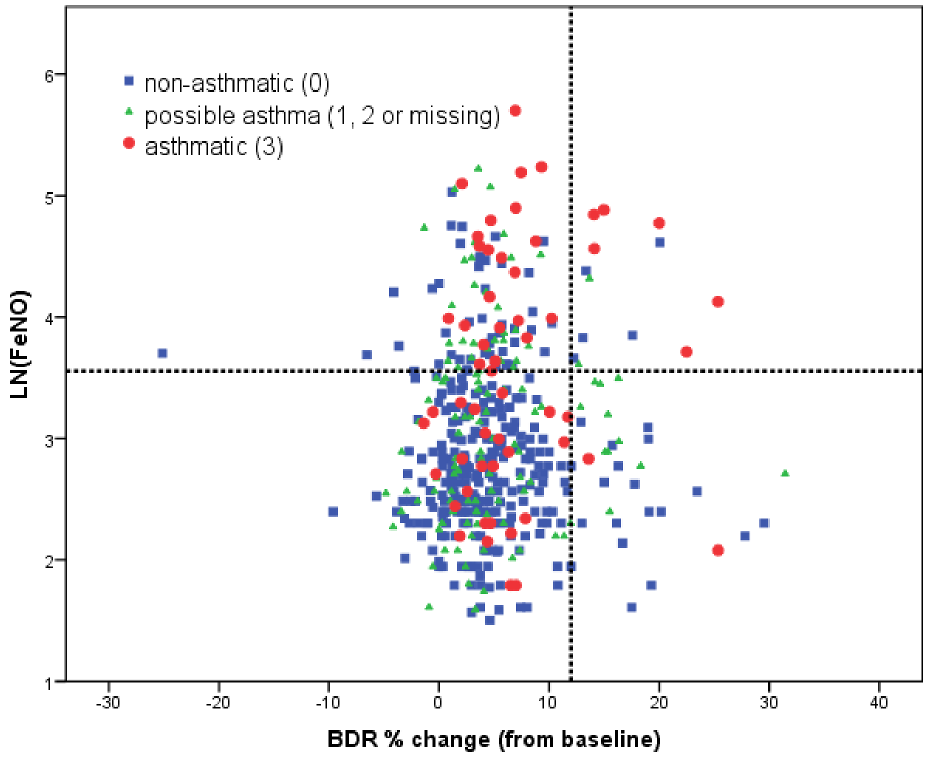

FEV_1/FVC

The measured mean FEV_1/FVC was very similar to predicted values for girls (89.7% Fig 4a and 89.5%, Fig 4b) and boys (86.9% Fig 4 c and 86.3% Fig 4 d). Only 10 children (1.6%, 3 girls) had $FEV_1/FVC<70\%$; of these, only 2 (1 girl) met our definition of asthma, 5 were non-asthmatic, and 3 with possible asthma.

Figure 4. FEV_1/FVC: a) Girls, measured, b) Girls, predicted c) Boys, measures d) Boys, predicted.

The measured mean FEV_1/FVC was very similar to predicted values for girls (89.7% and 89.5%, respectively) and boys (86.9% and 86.3% respectively E4), but showed a broader range.

a)

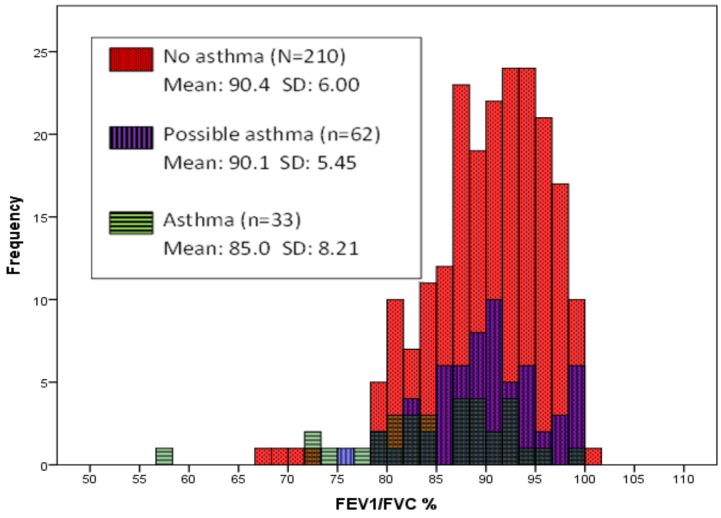

b)

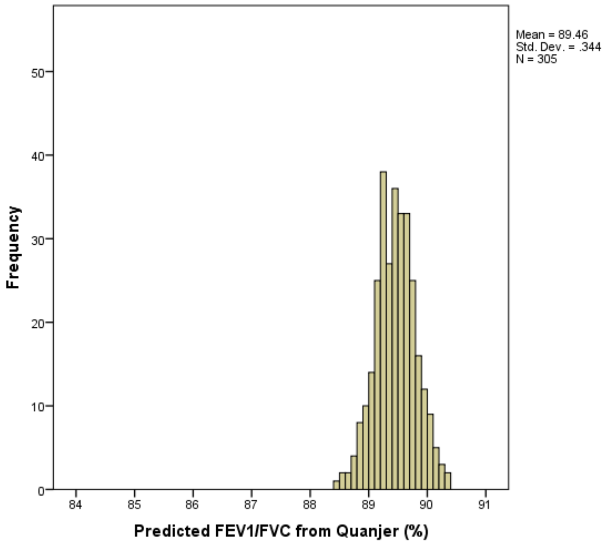

c)

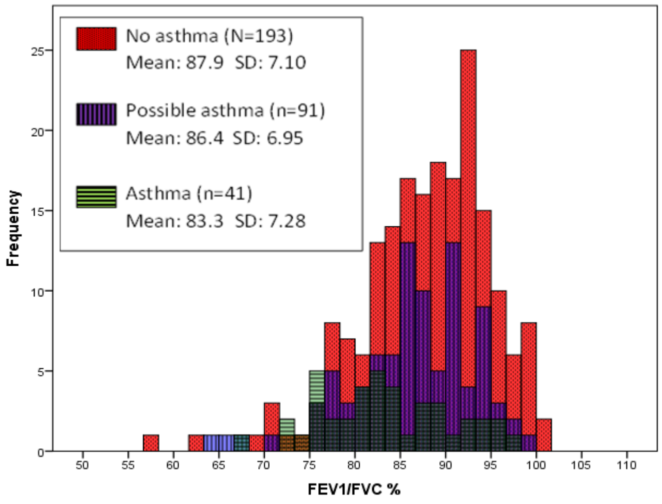

d)

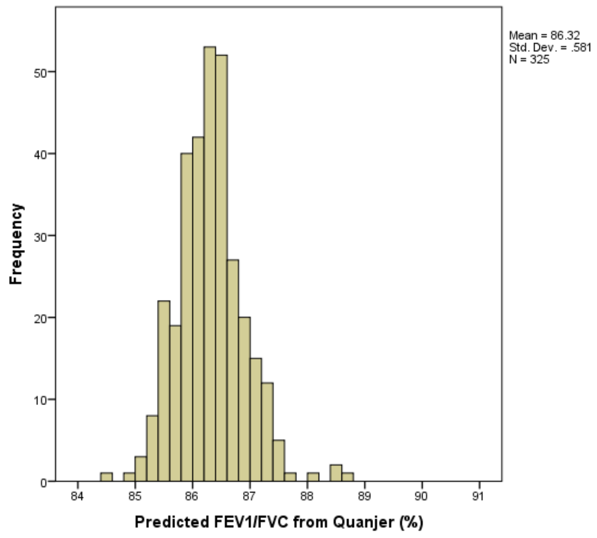

We calculated the LLN for FEV_1/FVC for each child; mean LLN was 78.2% for girls (Figure 5a) and 74.8% for boys (Figure 5b). Of the 28 children (4.4%, 11 girls) with FEV_1/FVC below LLN, 11 had asthma (6 girls), 5 had possible asthma (1 girl) and 12 were non asthmatic (4 girls).

Figure 5. Calculated LLN for FEV_1/FVC; a) girls, b) boys

a)

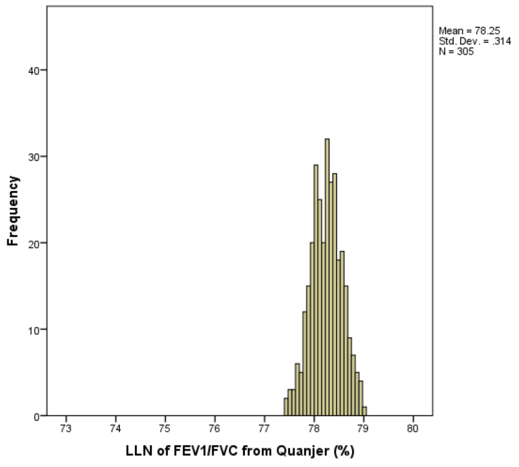

b)

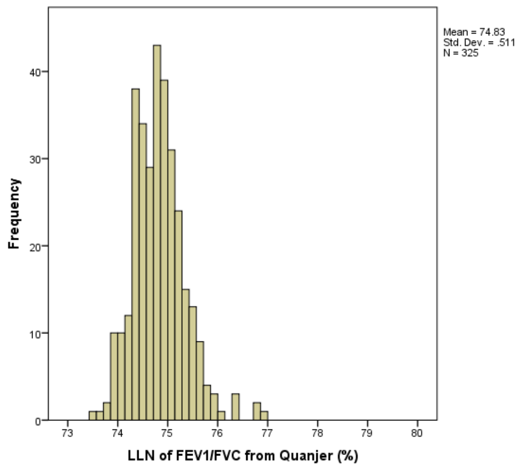

Figure 6. LLN for FEV_1/FVC across ages;

a) In Children, boys and for girls separately.

Height data taken from http://www.rcpch.ac.uk/child-health/research-projects/uk-who-growth-charts/uk-growth-chart-resources-2-18-years/school-age

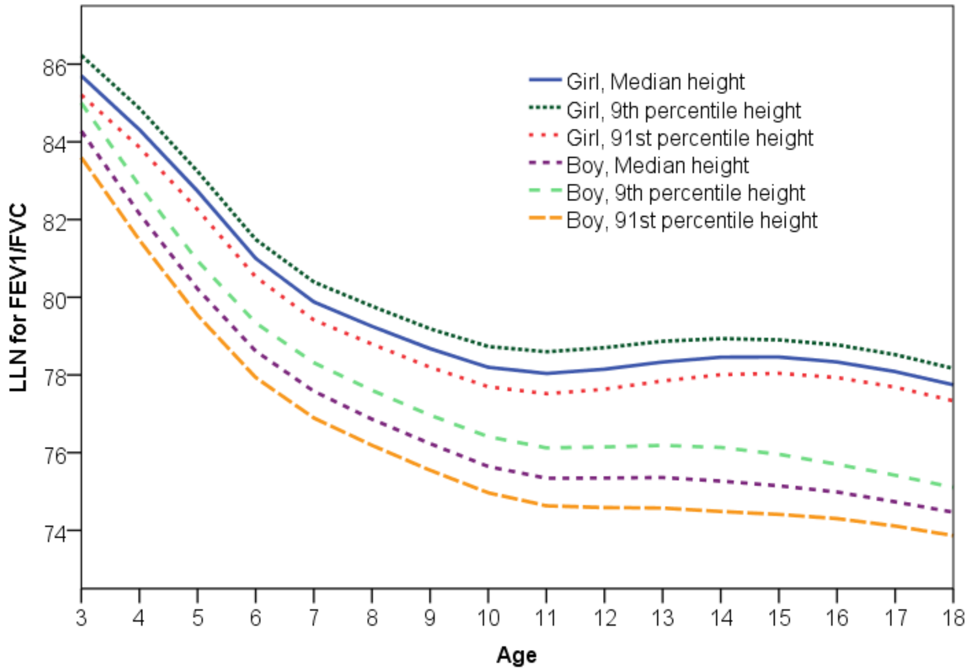

b) In adults, males and females shown separately; 2 heights used for illustrative purposes.

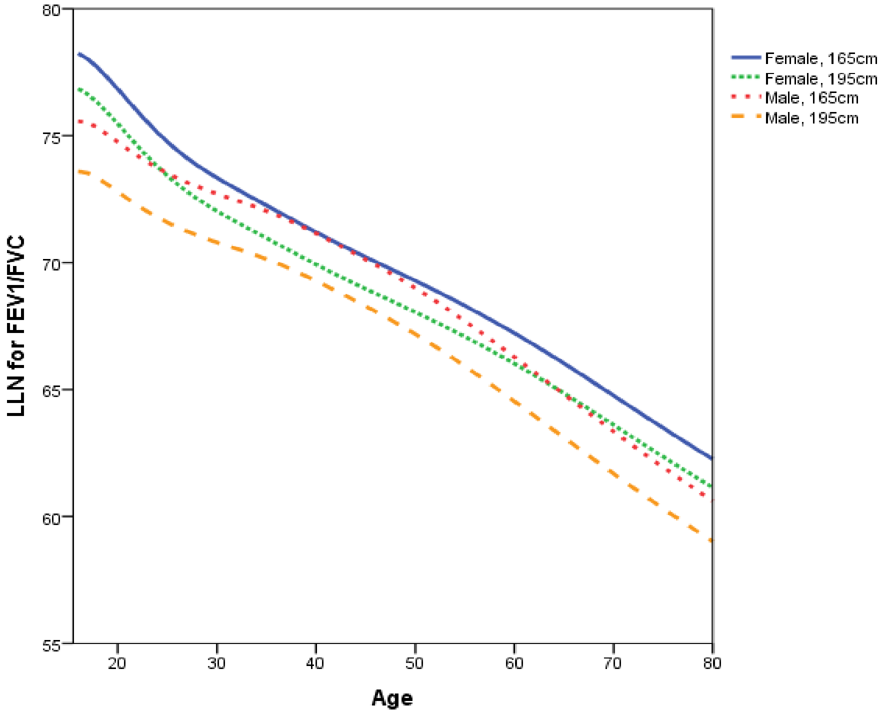

BDR

BDR did not differ significantly between boys and girls (p=0.21, Table 2). An increase in FEV_1 of ≥12% from baseline was observed in 54 children (8.7%), all of whom showed an improvement of 200mls or more. Of the 54 children with BDR≥12%, 12 had asthma (8 girls), 16 had possible asthma (5 girls), and 26 were non asthmatic (12 girls).

FeNO

FeNO was significantly higher in boys than girls (ppb, GM [95%CI]: 22.51 [20.45-24.79] vs. 17.70 [16.00-19.57], p=0.001, Table 3). FeNO was higher in children with rhinitis, but subgroup analysis revealed that this was only significant in those who did not have asthma (Tables 4 and 5). FeNO was ≥35ppb in 115/485 of children (23.7%), of whom 29 had asthma (14 girls), 32 had possible asthma (12 girls), and 54 were non asthmatic (22 girls).

Table 4. FeNO in study participants with and without rhinitis (n=461)

	FeNO GM (95% CI) ppb	Overall p-value	Pairwise p-values (Scheffé adjusted)		
			Vs 1	Vs 2	Vs 3
**No rhinitis (1) (n=279)**	17.49 (15.97-19.17)	<0.001	-	-	-
**Rhinitis, but no grass or birch sensitisation (2) (n=32)**	17.37 (12.99-23.23)		>0.99	-	-
**Rhinitis and grass and/or birch sensitisation; FeNO outside season (3) (n=78)**	24.09 (20.68-28.07)		0.012	0.23	-
**Rhinitis and grass and/or birch sensitisation; FeNO in season (4) (n=72)**	31.20 (26.42-36.85)		<0.001	0.004	0.22

Table 5. FeNO in children with asthma (n=51), with and without rhinitis

	FeNO GM (95% CI) ppb	Overall p-value*
**No rhinitis (n=18)**	42.46 (24.86-72.50)	0.74
**Rhinitis but no grass or birch sensitisation (n=8)**	26.81 (9.51-75.59)	
**Rhinitis and grass and/or birch sensitisation; FeNO outside season (n=12)**	39.77 (24.16-65.46)	
**Rhinitis and grass and/or birch sensitisation; FeNO in season (n=13)**	37.01 (22.19-61.71)	

*Pairwise p-values not calculated due to the lack of overall significance

Table 6. FeNO in those without asthma (n=300), with and without rhinitis

	FeNO, GM (95% CI) ppb	Overall p-value	Pairwise p-values (Scheffé adjusted)		
			Vs 1	Vs 2	Vs 3
**No rhinitis (1) (n=204)**	15.46 (14.14-16.90)	<0.001	-	-	-
**Rhinitis but no grass or birch sensitisation (2) (n=15)**	15.60 (11.36-21.43)		>0.99	-	-
**Rhinitis and grass and/or birch sensitisation; FeNO outside season (3) (n=43)**	22.05 (18.07-26.89)		0.014	0.36	-
**Rhinitis and grass and/or birch sensitisation; FeNO in season (4) (n=38)**	28.07 (22.55-34.94)		<0.001	0.032	0.42

Table 7. Multivariable model for prediction of asthma for whole population

	Odds ratio (95% CI)	p-value
Whole population (n=366)		
**FeNO (per ppb increase)**	1.03 (1.02, 1.04)	<0.001
**FEV_1/FVC (per % decrease)**	1.10 (1.04, 1.16)	0.001
**% change in FEV_1 after BDR test (per % increase)**	1.00 (0.94, 1.07)	0.97

Whole population, BDR removed		
**FeNO (per ppb increase)**	1.03 (1.02, 1.04)	<0.001
**FEV_1/FVC (per % decrease)**	1.10 (1.05, 1.15)	<0.001

	Odds ratio (95% CI)	p-value
Whole population (n=361)		
**FeNO (per ppb increase)**	1.02 (1.01, 1.03)	<0.001
**FEV_1/FVC (per % decrease)**	1.10 (1.04, 1.16)	0.001
**% change in FEV_1 after BDR test (per % increase)**	1.01 (0.94, 1.09)	0.72
**Atopy (yes vs no)**	2.70 (1.20, 6.07)	0.016

Whole population, BDR removed		
**FeNO (per ppb increase)**	1.02 (1.01, 1.03)	<0.001
**FEV_1/FVC (per % decrease)**	1.10 (1.05, 1.16)	<0.001
**Atopy (yes vs no)**	2.64 (1.19, 5.87)	0.017

Table 8. Summary of AUROC for measures of lung function used as continuous and dichotomous variables, based on the whole sample, but excluding those with possible asthma

Variable	Numbers of subjects Asthma : not Asthma	AUROC (95% CI)
**FEV_1/FVC % (as a continuous variable)**	74:403	0.701 (0.635-0.767)
**FEV_1/FVC < 70%**	74:403	0.507 (0.435-0.580)
**FEV_1/FVC < LLN**	74:403	0.559 (0.484-0.635)
**FEV_1/FVC < 83.8% ('best' cut-off)**	74:403	0.677 (0.605-0.749)
**FeNO ppb (as a continuous variable)**	56:314	0.711 (0.627-0.795)
**FeNO ≥ 35ppb**	56:314	0.673 (0.590-0.756)
**FeNO ≥ 24ppb ('best' cut-off)**	56:314	0.676 (0.596-0.755)
**BDR % (as a continuous variable)**	74:399	0.636 (0.568-0.705)
**BDR ≥ 12%**	74:399	0.548 (0.474-0.623)
**BDR ≥ 3.48% ('best' cut-off)**	74:399	0.612 (0.546-0.678)
**Probabilities from logistic regression (FeNO, FEV_1/FVC and BDR)**	56:310	0.787 (0.720-0.855)
**Probabilities from logistic regression (FeNO and FEV_1/FVC)**	56:310	0.787 (0.720-0.855)

Diagnosing asthma in children with recent symptoms

Of the 481 children with spirometry, BDR and FeNO measures available, 189 reported symptoms of cough or wheeze in the last 12 months

Table 9. Multivariable model for prediction of asthma – for children with symptoms

Children with symptoms, not on ICS (n=89)	OR (95% CI)	P value
**FeNO (per ppb increase)**	1.02 (1.01, 1.04)	0.006
**FEV_1/FVC (per % decrease)**	1.06 (0.98, 1.14)	0.17
**% change in FEV_1 after BDR test (per % increase)**	1.00 (0.89, 1.11)	0.94

Children with symptoms, not on ICS, BDR removed		
**FeNO (per ppb increase)**	1.02 (1.01, 1.04)	0.006
**FEV_1/FVC (per % decrease)**	1.06 (0.99, 1.12)	0.096

Children with symptoms, not on ICS (n=88)	**OR (95% CI)**	**P value**
**FeNO (per ppb increase)**	1.02 (1.00, 1.04)	0.006
**FEV_1/FVC (per % decrease)**	1.06 (0.97, 1.15)	0.19
**% change in FEV_1 after BDR test (per % increase)**	0.99 (0.89, 1.12)	0.92
**Atopy (yes vs no)**	1.98 (0.66, 5.94)	0.22

Children with symptoms, not on ICS, BDR removed		
**FeNO (per ppb increase)**	1.02 (1.00, 1.04)	0.025
**FEV_1/FVC (per % decrease)**	1.05 (0.99, 1.13)	0.11
**Atopy (yes vs no)**	1.99 (0.66, 5.95)	0.22

Table 10. Summary of AUROC for measures of lung function used as continuous and dichotomous variables, based on children with recent symptoms, but excluding those with possible asthma

Variable	Numbers of subjects Asthma : not Asthma	AUROC (95% CI)
**FEV_1/FVC % (as a continuous variable)**	34:55	0.616 (0.496-0.735)
**FEV_1/FVC < 70%**	34:55	0.482 (0.359-0.605)
**FEV_1/FVC < LLN**	34:55	0.522 (0.397-0.648)
**FEV_1/FVC < 85.5% ('best' cut-off)**	34:55	0.625 (0.504-0.746)
**FeNO ppb (as a continuous variable)**	34:55	0.618 (0.487-0.750)
**FeNO ≥ 35ppb**	34:55	0.639 (0.516-0.761)
**FeNO ≥ 37ppb ('best' cut-off)**	34:55	0.648 (0.526-0.770)
**BDR % (as a continuous variable)**	34:55	0.594 (0.475-0.713)
**BDR ≥ 12%**	34:55	0.508 (0.383-0.632)
**BDR ≥ 3.2% ('best' cut-off)**	34:55	0.606 (0.487-0.725)
**Probabilities from logistic regression (FeNO, FEV_1/FVC and BDR)**	34:55	0.682 (0.565-0.799)
**Probabilities from logistic regression (FeNO and FEV_1/FVC)**	34:55	0.683 (0.567-0.800)

Text relating to Figure 2b: FEV_1/FVC<LLN in main manuscript:

Eight of the 89 children had obstructive spirometry using LLN criteria, five of whom had BDR≥12%, meeting the criteria for asthma. Three of these children met our definition of asthma. All three remaining children with BDR<12% had FeNO<35ppb, which would trigger a referral for specialist assessment. Among 81 children with FEV_1/FVC≥LLN, 22 had FeNO≥35ppb and would be diagnosed with asthma, or suspected asthma depending on the results of PEFR monitoring; 13 of these children met our criteria for asthma. FeNO was <35ppb in 59 children (17 of whom met our criteria for asthma), and if a PEF diary had shown 20% reversibility would fall in to the suspect asthma part of the algorithm, in which tests should be repeated at 6 weeks.

Figure 7. Diagnostic algorithm, starting with 163 children with respiratory symptoms; 34 of whom had asthma, 55 did not have asthma and 74 had possible asthma. The number in parenthesis denotes the number of children with this test result who had asthma. PEF variability was not available in this population.

a) Using FEV_1/FVC<70% to denote obstructive spirometry

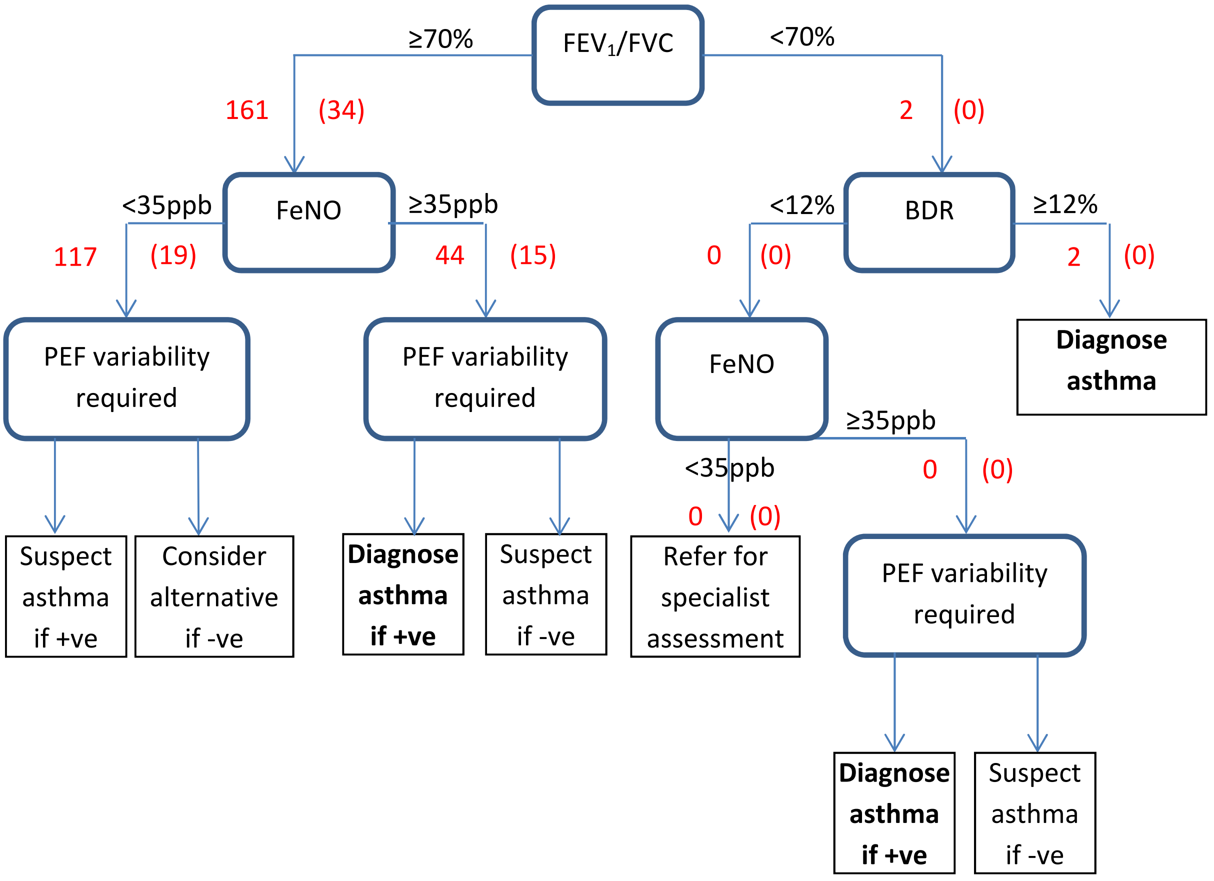

b) Using FEV_1/FVC<LLN to denote obstructive spirometry

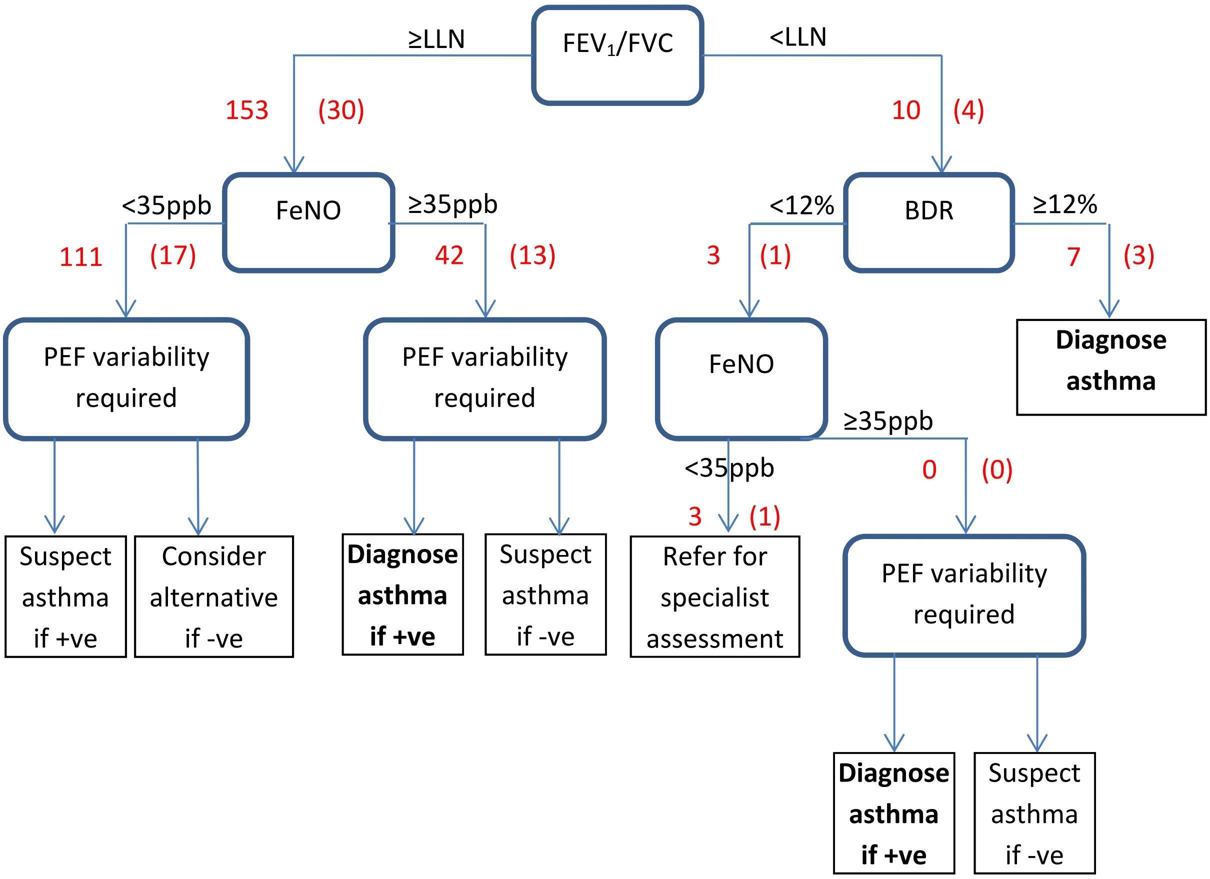

References

1. Reshef, D. N., Reshef, Y. A., Finucane, H. K., Grossman, S. R., McVean, G., Turnbaugh, P. J., Lander, E. S., Mitzenmacher, M., and Sabeti, P. C. (2011) Detecting novel associations in large data sets. Science 334, 1518-1524
